# Risk of weaning failure with PAOP ≥15 versus PAOP ≥18

**DOI:** 10.1186/cc12086

**Published:** 2013-03-19

**Authors:** Y Nassar, A Abdelbary

**Affiliations:** 1 Cairo University, Giza, Egypt

## Introduction

Despite 18 mmHg being recognized as a classical cutoff value, there is no definite value of PAOP above which cardiogenic oedema develops. We aimed to assess the risks of weaning failure with PAOP ≥15 and with PAOP ≥18.

## Methods

A prospective enrollment of 30 adult medical ICU patients on invasive mechanical ventilation who were fulfilling the criteria for initiation of weaning from mechanical ventilation according to the Sixth International Conference Statement of Intensive Care Medicine. We followed the two-step weaning strategy, which involves assessment regarding readiness for weaning followed by a spontaneous breathing trial (SBT) as a diagnostic test to determine the likelihood of successful extubation. Invasive right heart catheterization to measure PAOP, and non-invasive transthoraxic echo and tissue Doppler parameters E/E', E/A, DT, IVRT were measured before initiation and after termination of the SBT.

## Results

E/E' was significantly correlated with PAOP (*r *= 503, *P <*0.001). A cutoff value of septal E/E' >11.3 predicted PAOP elevation ≥18 mmHg with a sensitivity of 90.9% and specificity of 87.8%, while a value of septal E/E' >10.9 predicted PAOP elevation ≥15 mmHg with a sensitivity of 59.1% and specificity of 87.8%. The incidence of weaning failure was 23%, and 72% of patients who failed to be weaned exhibited significantly higher PAOP during the trial. Patients with PAOP ≥18 at the end of the SBT mostly (72.4%) failed weaning while a minority (27.6%) successfully weaned. The majority (91.3%) of the patients with PAOP <18 had a successful weaning outcome and 8.7% failed weaning outcome. PAOP ≥18 significantly negatively correlated with weaning success (*P *= 0.001, *r *= -0.627). On the other hand, PAOP ≥15 did not show a significant correlation (*P *>0.05, *r *= -0.274). The PAOP ≥18 weaning failure risk estimate was 8.2, with 95% CI 0.004 to 0.3. The PAOP ≥15 weaning failure risk estimate was 1.83, with a significant 95% CI of 0.913 to 3.648.

## Conclusion

E/E' was significantly correlated with PAOP (*r *= 503, *P <*0.001). E/E' > 11.3 predicted PAOP elevation ≥18 mmHg (sensitivity 90.9% and specificity 87.8%), while E/E' >10.9 predicted PAOP elevation ≥15 mmHg (sensitivity 59.1% and specificity 87.8%). Weaning failure risk is greater (8.2 vs. 1.8) with PAOP ≥18 versus PAOP ≥15, respectively.

